# Impact of Postponement of Appointments on Vision and Psychological Well-Being Among Outpatients Attending Ophthalmology Clinics: A Malaysian Perspective

**DOI:** 10.7759/cureus.38423

**Published:** 2023-05-02

**Authors:** Bannu Jayallan, Nor Fariza Ngah, Nurul Ilham Hussain, Nik Ruzyanei Nik Jaafar, Azimatun Noor Aizuddin, Meng Hsien Yong, Norshamsiah Md Din, Mae-Lynn Catherine Bastion

**Affiliations:** 1 Department of Ophthalmology, Hospital Canselor Tunku Mukhriz Universiti Kebangsaan Malaysia, Kuala Lumpur, MYS; 2 Department of Ophthalmology, Hospital Shah Alam, Selangor, MYS; 3 Department of Psychiatry, Hospital Canselor Tunku Mukhriz Universiti Kebangsaan Malaysia, Kuala Lumpur, MYS; 4 Department of Research and Statistics, Hospital Canselor Tunku Mukhriz Universiti Kebangsaan Malaysia, Kuala Lumpur, MYS

**Keywords:** psychology, vision, postponement, appointment, ophthalmology, covid-19

## Abstract

Introduction: During the COVID-19 pandemic, non-frontline medical disciplines, including ophthalmology, were advised to minimize their services to channel crucial healthcare resources to manage the surge in COVID-19 cases. The ophthalmology department postponed all non-urgent appointments and elective surgical procedures. However, little is known about the visual and mental health impact of these changes in ophthalmology services. Therefore, our study aimed to explore the impact of postponement in ophthalmology outpatient clinic appointments towards visual acuity (VA) changes and the psychological well-being of patients during the COVID-19 pandemic in Malaysia.

Methodology: This cross-sectional study, utilizing a convenience sampling method, recruited patients attending ophthalmology outpatient clinic services from July 2020 to June 2021 to participate in the study. The Snellen chart was used to measure the VA, and the Kessler psychological distress scale (K-10) was used to measure psychological distress levels among patients with (study) and without (controls) postponement of the appointment.

Results: A total of 485 patients were included in the data analysis; 267 study and 218 controls. There is a statistically significant difference in categorical change of VA (p < 0.001) and categorical K-10 score (p = 0.048) among the study and control groups. Nonetheless, a decline in VA alone does not show a statistically significant association with an increased probability of experiencing psychological distress (p=0.149).

Conclusion: Postponement of ophthalmology appointments negatively affected the VA and the psychological well-being of patients. Appropriate assessment of patients before postponing their appointment is crucial to mitigate the worsening of VA and psychological distress.

## Introduction

The coronavirus disease 2019, which is also widely known as COVID-19, remains a Public Health Emergency of International Concern (PHEIC) [1]. To date, as of 31st December 2022, there are a total of 730 763 167 cases and 6 697 328 deaths globally [2]. The World Health Organization (WHO) has outlined public health and social measures to reduce the spread of COVID-19 at the community, national, and international levels. Many countries around the globe implemented lockdowns to curb the rise in COVID-19 cases; Malaysia was no exception. The Malaysian government announced its first movement control order (MCO) starting on 18 March 2020. With the prolonged MCO, health concerns, psychological distress, and economic implications were unavoidable [3].

During the peak of the COVID-19 infection, the healthcare system in many countries, including developed countries like the United States of America and European nations, was overstretched and on the brink of collapse. Therefore, drastic measures were taken at ministerial and hospital levels to cope with the surge in COVID-19 cases, reduce the transmission of the virus, and conserve medical supplies for emergency departments and intensive care units. Other medical disciplines, including ophthalmology that were not directly involved in the management of COVID-19 patients were advised to minimize their services to channel crucial healthcare resources to manage the surge in COVID-19 cases [4].

Ophthalmology staff are referred to as a higher-risk group because they have close contact with patients during an ocular examination, for instance, the slit-lamp examination. In addition, ophthalmology outpatient clinics tend to have a high number of daily outpatient visits and overcrowded waiting rooms [5]. This drew the attention of many international ophthalmology societies, including the Malaysian Society of Ophthalmology (MSO), which recommended avoiding any treatment other than urgent or emergent care [6]. These recommendations were also in keeping with many of the hospital management decisions, including those of the study centers in our research, which were Hospital Canselor Tuanku Muhriz Universiti Kebangsaan Malaysia (HCTM) and Hospital Shah Alam (HSA). All non-urgent outpatient specialty clinic appointments, including the Ophthalmology clinic, were rescheduled starting 18 March 2020. The specialty clinics were closed to elective and scheduled appointments.

To fulfill the recommendation to reduce specialty clinic cases, it was crucial to assess the risk and postpone non-urgent outpatient appointments and elective surgical procedures [7,8]. The doctors in charge of managing the caseload at the clinic briefly screened all the patients’ case notes before deciding to reschedule their appointment. The doctors contacted the patients via phone or text message to inform them of the clinic’s closure and give them a new appointment date. During the earlier phase of the COVID-19 pandemic, the clinic was only receiving emergency cases and urgent referrals from other departments.

A few studies have been conducted internationally to investigate the correlation between visual acuity and scheduled hospital visit adherence. A study conducted before the COVID-19 pandemic has supported the idea that delay in ophthalmology outpatient clinic follow-up has caused visual impairment in certain ophthalmological diseases such as neovascular age-related macular degeneration (AMD), retinal vein occlusions (RVO), diabetic macular edema (DMO), rhegmatogenous retinal detachment (RRD), as well as glaucoma [9]. A multicenter study on the impact on visual acuity in neovascular age-related macular degeneration in Europe due to the rescheduling of appointments during the COVID-19 pandemic also reported significant visual loss post-lockdown [10].

In addition to the physical complications, postponement of appointments also causes psychological distress, a complication that is often overlooked. Psychological distress can be explained as emotional distress associated with a stressor. Public anxiety and stigma towards COVID-19 have increased the prevalence of mental health illnesses such as anxiety and depression. According to the American Psychological Association (APA), anxiety is an emotion characterized by feelings of tension and excessive worries associated with negative thoughts and physiological changes, whereas depression is a prolonged feeling of sadness and/or loss of interest in a previously pleasurable activity. Depression also affects patients’ biological symptoms, such as appetite and sleep, as well as their ability to function and interpersonal relationships [11].

Various research done locally and internationally has shown a rise in psychological distress in the community and among healthcare staff during the COVID-19 pandemic [4,12-13]. Patients with chronic health conditions, including many eye diseases, are at a higher risk of psychological distress. The alarming rise in psychological distress worldwide has drawn the attention of the medical fraternity to the heavy load of COVID-19 on mental health, which may have changed how people view the impact of COVID-19. Delay or postponement of clinic appointments has a direct negative effect on the mental health of patients [14].

There are very few studies on the impact of the COVID-19 pandemic on the outpatient healthcare system among Ophthalmology patients. To the best of our knowledge, there is no local study exploring both the physical and mental health impacts of changes in ophthalmology outpatient clinic services in Malaysia. Hence, our study aims to explore the association between the postponement of ophthalmology outpatient clinic appointments and visual acuity changes, as well as its correlation to psychological distress during the COVID-19 pandemic in Malaysia.

## Materials and methods

This cross-sectional study, utilizing a convenience sampling method, recruited patients attending ophthalmology outpatient clinic services at HCTM and HSA from July 2020 to June 2021 to participate in the study. HCTM is a teaching hospital located in Kuala Lumpur, whereas HSA is a tertiary public hospital located in Selangor. Both Kuala Lumpur and Shah Alam were the epicenters of the COVID-19 pandemic in Malaysia. Since the pandemic started, the Malaysian government has designated HCTM and HSA as hybrid hospitals to manage COVID-19 cases in Malaysia. HCTM and HSA are tertiary referral centers for ophthalmology services. The study duration of July 2020 to June 2021 was selected as the outpatient clinics gradually increased their number of patients and services offered during this period. 

Patients that presented to the Ophthalmology outpatient clinics in HCTM and HSA during the study period were invited to participate in the study. Informed consent was obtained from all the participants. A total of 510 patients voluntarily consented to participate in the study after reviewing the inclusion and exclusion criteria.

The participants who agreed to participate in the study were given the Kessler psychological distress scale (K-10) questionnaire to complete while waiting for their consultation with the attending doctor. All participants were also given pamphlets on stress management and contact details for psychological support centers. The researchers encouraged participants who scored moderate or severe psychological distress on the K10 questionnaire to contact the principal investigator or visit the nearest healthcare facility for further evaluation and treatment. The patient information sheet, consent form, and K-10 questionnaire were made available in both Malay and English. The pamphlets on stress management were made available in Malay, English, and Mandarin. The forms and questionnaires were given according to the participants’ language preferences.

Participants who completed the K-10 questionnaire returned the questionnaire to the attending doctor. The required demographic data, clinical assessment, and visual acuity were filled out on the data collection sheet by the attending doctors. Participants who completed the questionnaire were screened once again by the researchers based on inclusion and exclusion criteria. Twenty-five patients who did not meet the inclusion criteria or met the exclusion criteria were excluded from this study. Therefore, only 485 participants who voluntarily consented to participate in the study, met the inclusion criteria and did not fulfill the exclusion criteria were included in the study analysis. The data from the data collection sheet was transferred into an MS Excel spreadsheet (Redmond, USA).

Inclusion and exclusion criteria 

The inclusion criteria for the study group were patients aged 18 years and above who had at least one postponed appointment under the Ophthalmology clinic due to MCO during the COVID-19 pandemic in the preceding months and were attending their postponed appointment for the first time. The inclusion criteria for the control group were patients aged 18 years and above who attended their regular follow-up and who did not have postponed appointments in the Ophthalmology outpatient clinic during the MCO. Participants who canceled their appointment on their own accord missed their scheduled appointment, had a known underlying psychiatric illness, or were COVID-19 positive before the appointment were excluded from the study.

Measurement tools

A data collection sheet was designed for the study to collect the patients' social demographic data, appointment data, visual acuity data, and psychological data. Participants’ visual acuity was assessed using the Snellen chart and later converted to LogMAR for analysis. Participants’ psychological data was assessed using the Kessler psychological distress scale (K-10). Participants were given the option to answer the questionnaire in either Malay or English.

Visual Acuity in LogMAR

Vision is a complex integration of light, form, contrast, and color sense. Visual acuity (VA) is referred to as the spatial limit of visual discrimination. VA is defined as the minimum resolvable visual angle measured in minutes of arc for a standard test pattern. The Snellen Chart was used to determine the smallest letter the patient could read from six meters away. LogMAR is the logarithm of the minimum angle of resolution that uses decimal points to indicate the smallest letter the patient can read. The outpatient ophthalmology clinic in both study centers used a Snellen Chart to record VA. Only the eye with the worst VA pre-MCO of each participant was selected for the study. If both eyes had the same VA pre-MCO, the left eye was selected for analysis. The VA data was then converted to a standardized logMAR format for statistical analysis. The change in VA was calculated by logMAR post-MCO subtracting logMAR pre-MCO. Lower logMAR scores signify better visual acuity. The change in VA based on logMAR scores in both the study and control groups was further categorized into improvement, no change, and worsening of VA to make it clinically relevant [15].

Kessler Psychological Distress Scale (K-10) Malay and English Version 

A validated K-10 questionnaire, comprising a total of 10 questions, was used as a screening tool to measure the psychological distress level of participants. Each question is scored on a scale of one to five. The total scores of each question will be added, and the sum of the scores will be categorized further as “0 - 19” likely to be well, “20 - 24” likely to have a mild disorder, “25 - 29” likely to have a moderate disorder, and “30 - 50” likely to have a severe disorder [16]. Both the English and Malay versions were validated in Malaysia. The translated Malay version of this scale has a Cronbach’s alpha of 0.885 among patients with psychological distress [17]. In this study, participants with a score of 19 and below were categorized as “likely psychologically not in distress,” whereas participants with a score of 20 and above were categorized as “likely psychologically distressed” [16,18].

Statistical analyses 

IBM Corp. Released 2020. IBM SPSS Statistics for Windows, Version 27.0. Armonk, NY: IBM Corp was used for data analysis. A descriptive statistic was carried out to summarize the independent and dependent variables. Normality testing was conducted through a combination of both statistical (continuous variables, with values of skewness and kurtosis less than 2.00 considered to be normally distributed) and graphical (boxplots) modalities. Continuous data were presented as mean and standard deviation if the data were normally distributed, whereas median and interquartile range were used if the data were not normally distributed. Categorical variables were presented as frequency and percentage. The differences in the various characteristics of the participants between the postponed appointment group (study group participants) and the non-postponed appointment group (control group participants) were compared using the independent sample t-test or Pearson chi-squared test, whichever was appropriate. The changes in visual acuity and K-10 scores among the study and control groups were compared using the Pearson Chi-square test. The Pearson Chi-square test was also used to explore the correlation between the change in visual acuity among the "psychologically distress” and “not in psychological distress” groups according to the K-10 scores. All the tests were two-sided, and statistical significance was denoted as p<0.05. 

Ethical considerations 

All patients who participated in the study were given a patient information sheet before being recruited. They voluntarily gave their consent to participate in the study. No identifiable details such as name, email, or contact number were collected from the patients to ensure anonymity. The patients who were psychologically distressed with a score of “moderate to severe” in the K-10 questionnaire were offered further evaluation with a referral to the psychiatry team. All patients were given contact details for psychological support centers. The study was conducted according to the guidelines of the Declaration of Helsinki. This study was approved by the Research Ethics Committee of the National University of Malaysia (UKM PPI/111/8/JEP-2020-430) and the Medical Research and Ethics Committee, Ministry of Health Malaysia [NMRR-20-1123-54953 (IIR)]. 

## Results

A total of 485 patients attending the Ophthalmology outpatient clinic in HCTM and HSA were included in the data analysis. A combination of statistical and graphical normality tests showed continuous data for age, change in VA (logMAR), and K-10 scores were normally distributed, whereas the delay in days of outpatient appointments violated the normal distribution. Table 1 displays the demographic data of the study and control group participants. The mean age among the study and control groups was similar: 62 years old. The majority of the participants in the study and control group were male and of Malay ethnicity. Most of the participants’ employment status was not negatively affected during the COVID-19 pandemic. Only 15 (6.88%) participants in the control group and 17 (6.37%) in the study group reported that their employment was negatively affected during the COVID-19 pandemic. There was no significant difference in age, gender, ethnicity, or change in employment status observed between the study and control groups (p > 0.05, Table 1). The median delay of outpatient clinic appointments in the study group was 120 days.

**Table 1:** Demographic data of the study and control group. n: number of patients;  SD: standard deviation ^a^Independent t-test; ^b^Pearson chi-square test

Demographic data	Study group (n=267)	Control group (n=218)	p-value
Mean age in years: mean (SD)	61.84 (14.28)	61.56 (14.50)	0.832^a^
Gender: n (%)			
Male	139 (52.06)	120 (55.05)	0.512^b^
Female	128 (47.94)	98 (44.95)	
Ethnicity: n (%)			
Malay	151 (56.56)	131 (60.09)	0.547^b^
Chinese	78 (29.21)	52 (23.85)	
Indian	36 (13.48)	32 (14.68)	
Others	2 (0.75)	3 (1.38)	
Change in employment status: n (%)			
Yes, negatively affected	17 (6.37)	15 (6.88)	0.821^b^
No	250 (93.63)	203 (93.12)	

The mean change in VA (logMAR) was 0.05 (SD = 0.22) in the study group and -0.02 (SD = 0.21) in the control group (p = 0.001), whereas the mean K-10 score was 20.15 (SD = 4.86) in the study group and 19.15 (SD = 3.24) in the control group (p = 0.007). Table 2 shows the categorical change in VA and K-10 scores among the study and control groups. Despite the percentage of participants experiencing ‘no change in VA’ being similar between the study group (63%) and the control group (65%), there was a considerable difference in participants experiencing improvement and worsening in visual acuity between the two groups. In the study group, only 13% experienced an 'improvement of VA’ and 24% experienced a ‘worsening of VA’, whereas in the control group, 23% experienced an ‘improvement of VA’ and 12% experienced a ‘worsening of VA’. There is a statistically significant difference in categorical change in VA among the study and control groups (p < 0.001). There was a greater percentage of participants in the study group (34%) that were ‘likely psychologically distressed’ compared to the control group (26%). There is a statistically significant difference in categorical K-10 scores among the study and control groups (p = 0.048).

**Table 2:** Change in visual acuity and K-10 score among study and control group. n: number of patients; VA: visual acuity; K-10: Kessler psychological distress scale ^a^Pearson chi-square test

Variables	Study group n (%)	Control group n (%)	p-value
Change of visual acuity (VA)			
No change of VA	169 (63.30)	142 (65.14)	<0.001^a^
Improvement of VA	35 (13.10)	50 (22.94)	
Worsening of VA	63 (23.60)	26 (11.93)	
K-10 score			
Likely psychologically not in distress	175 (65.54)	161 (73.85)	0.048^a^
Likely psychologically distressed	92 (34.46)	57 (26.15)	

Table 3 illustrates the correlation between changes in VA and psychological distress. Participants who were ‘likely psychologically distressed’ had a higher mean change in VA score (0.061) compared to those who were ‘likely psychologically not in distress’ (-0.002). There was a statistically significant difference in mean change in VA among participants that were ‘likely psychologically distressed’ compared to ‘likely psychologically not in distress’ (p = 0.013). However, when the mean change of VA was further categorized to justify clinical relevance, there was no statistically significant difference in categorical change in VA among participants that were ‘likely psychologically distressed’ compared to ‘psychologically not in distress’ (p = 0.149). Nonetheless, among the participants that were ‘likely psychologically distressed’, 23% had worsening vision compared to 16% among participants that were ‘psychologically not in distress’. 

**Table 3:** Correlation between change in visual acuity and psychological distress. n: number of patients;  SD: standard deviation; VA: visual acuity; logMAR: logarithm of the minimum angle of resolution ^a^Independent t-test; ^b^Pearson chi-square test

Change in Visual Acuity (logMAR)	Likely psychologically distressed (n=149)	Likely psychologically not in distress (n=336)	p-value
Change in visual acuity: mean (SD)	0.061 (0.28)	-0.002 (0.18)	0.013^a^
Change of visual acuity (VA): n (%)			
No change of VA	90 (60.40)	221 (65.77)	0.149^b^
Improvement of VA	24 (16.11)	61 (18.16)	
Worsening of VA	35 (23.49)	54 (16.07)	

## Discussion

This study revealed that the postponement of appointments due to the COVID-19 pandemic has negatively affected the VA outcomes of our patients visiting the ophthalmology outpatient clinic. While much emphasis in previous studies has been devoted to the physical health complications due to postponement of appointments, this study reveals that postponement of appointments also causes significant psychological distress. The psychological distress due to the postponement of the appointment is independent of the change in VA. Outpatient clinic appointment adherence is vital to the overall physical and psychological well-being of the patients. 

The findings of our research are in accordance with numerous previous studies done to investigate the impact of the loss of follow-up or postponement in patient care in the field of ophthalmology. Multiple studies done in other countries have shown that patients who had a delay in their appointment had significant vision loss due to the progression of disease activity, which led to a poorer long-term prognosis. Nonetheless, the reason for the postponement of these studies was the patient's own accord [9,19-21]. However, in our study, the appointments were postponed due to an unprecedented global pandemic, which required drastic measures such as outpatient clinic closure for all elective or scheduled appointments, except for urgent and emergency cases, to curb the rise in COVID-19 cases. This radical measure has ultimately affected ophthalmology patient care, leading to a reduction in VA. The worsening in VA may be due to the progression of the disease without adequate monitoring and halted treatment regimes [21]. Unfortunately, the delay in follow-up and care beyond clinically recommended intervals has been shown to have a permanent visual impairment that could result in significant morbidity for the patients [22]. 

In contrast, Alkharashi et al. reported no statistically significant differences between patients’ VA in both eyes before and after the lockdown among patients who had an appointment delay due to the COVID-19 lockdown. This study had strict criteria for the types of cases that were postponed and was further limited to cases that had mild to no visual impairment before the lockdown [23]. The enormity and uncertainty of the COVID-19 pandemic in Malaysia may have led to haphazard postponements of patients’ appointments. There were also instances where patients’ appointments were postponed multiple times due to the prolonged pandemic and the changes in hospital policy. At our study centers, patients that belong to the high-risk population of getting complications of COVID-19, which included vulnerable age groups and those with medical comorbidities, were prioritized to be postponed. However, we might have overlooked the fact that these groups might also be most vulnerable to suffering from worsening VA due to the postponement of the appointment. Therefore, to mitigate the long-term complications of the postponement of appointments, there should be strict criteria on-site when selecting cases to be postponed. 

Studies during the COVID-19 pandemic revealed that there was an increased prevalence of psychological distress, especially anxiety, and depression, in the general and hospital populations [24,25]. Various psychosocial factors could contribute to the worsening psychological distress, which includes being infected with COVID-19 [26]. Therefore, to reduce the bias of psychological distress associated with being infected by COVID-19, our study excluded participants who had been infected by COVID-19 before the appointment. Our study demonstrated that postponement of appointments was also among the factors that could cause psychological distress among ophthalmology outpatient clinic patients (p = 0.048). Despite patients with worsening VA having higher psychological distress, these findings were not statistically significant (p = 0.149). This intriguing finding from our study highlights that the postponement of appointments had a greater impact on psychological well-being compared to the VA post-MCO. 

A study conducted in the United Kingdom discussed the psychosocial impact of the COVID-19 lockdown among ophthalmology patients. Forty-six percent of the participants reported that the fear of further vision loss because of delayed review or treatment was among the factors that contributed to their psychological distress [27]. This demonstrates that postponement of the appointment itself could cause psychological distress among patients, irrespective of the physical complications. Nonetheless, there are also pre-pandemic studies that revealed that the worsening of VA, irrespective of the severity of VA change and the disease progression, caused psychological distress among patients. Hence, change in VA is also an important factor associated with the mental health status of an ophthalmology patient, irrespective of the ocular diagnosis [28]. 

To ensure patients are managed holistically, an ophthalmologist mustn't disregard the emotional well-being of their patients. In addition to focusing on the VA outcome of ophthalmology patients, addressing factors that could influence the mental health of patients can improve the quality of care provided and ultimately enhance patients’ quality of life. Therefore, the ophthalmology department must prepare appropriate strategies/guidelines for delaying outpatient appointments to mitigate the impact of the postponement on both the VA and mental health. 

There are a few limitations to this study. The study was limited to two tertiary hospitals with ophthalmology services in Malaysia. Therefore, the findings of the study population might not represent all the ophthalmology outpatients in Malaysia. The study’s cross-sectional design could only identify associations between variables and render the study of causality implausible. Furthermore, conducting the study during the COVID-19 pandemic also renders the findings of the study population less accurate during other periods when patients’ appointments are postponed. The study also did not include household income, other underlying medical illnesses, social life, or family demographics, which could be confounding factors for the psychological well-being of patients during the pandemic. 

## Conclusions

To the best of the authors’ knowledge, this is the first study evaluating the change in VA and the psychological impact due to the postponement of ophthalmology appointments during the COVID-19 pandemic in Malaysia. While much emphasis has been devoted to the change in VA among ophthalmology outpatients due to postponement of appointments, the present study establishes that postponement of appointments negatively impacted the VA and psychological well-being of ophthalmology outpatients at two major study centers in Malaysia. Among the intriguing findings of the study was that postponement of appointments had a greater impact on psychological distress compared to the VA post-MCO, highlighting the crucial impact of postponement of appointments on patients’ mental health. Thus, appropriate assessment and screening of patients before the postponement of appointments should be a key priority to mitigate the negative impact of the postponement on the VA and the psychological well-being of patients.
